# Altered functional connectivity of the ascending reticular activating system in obstructive sleep apnea

**DOI:** 10.1038/s41598-023-35535-4

**Published:** 2023-05-30

**Authors:** Jung-Ick Byun, Geon-Ho Jahng, Chang-Woo Ryu, Soonchan Park, Kun Hee Lee, Sung Ok Hong, Ki-Young Jung, Won Chul Shin

**Affiliations:** 1 Department of Neurology, Kyung Hee University College of Medicine, Kyung Hee University Hospital at Gangdong, Seoul, 05278 Republic of Korea; 2 Department of Radiology, Kyung Hee University College of Medicine, Kyung Hee University Hospital at Gangdong, Seoul, Republic of Korea; 3 Department of Otorhinolaryngology-Head and Neck Surgery, Kyung Hee University College of Medicine, Seoul, Republic of Korea; 4 Department of Oral and Maxillofacial Surgery, Kyung Hee University College of Dentistry, Kyung Hee University Dental Hospital at Gangdong, Seoul, Republic of Korea; 5 Department of Neurology, Neuroscience Research Institute, Seoul National University College of Medicine, Seoul National University Hospital, Seoul, 110-744 Republic of Korea

**Keywords:** Sleep disorders, Neural circuits

## Abstract

Repeated arousals during sleep in obstructive sleep apnea (OSA) may lead to altered functional connectivity (FC) of the ascending reticular activating system (ARAS). We evaluated resting-state FC between eight ARAS nuclei and 105 cortical/subcortical regions in OSA patients and healthy controls. Fifty patients with moderate to severe OSA and 20 controls underwent overnight polysomnography and resting-state functional magnetic resonance imaging. Seed-to-voxel analysis of ARAS–cortex FC was compared between OSA patients and controls. The ARAS nuclei included the locus coeruleus (LC), laterodorsal tegmental nucleus (LDTg), and ventral tegmental area (VTA). FC values of three ARAS nuclei (the LC, LDTg, and VTA) significantly differed between the groups. FC of the LC with the precuneus, posterior cingulate gyrus, and right lateral occipital cortex (LOC) was stronger in OSA patients than controls. FC between the LDTg and right LOC was stronger in OSA patients than controls, but FC between the VTA and right LOC was weaker. Average LC–cortex FC values positively correlated with the arousal, apnea, and apnea–hypopnea index in OSA patients. Alterations in ARAS–cortex FC were observed in OSA patients. The strength of LC–cortex noradrenergic FC was related to arousal or OSA severity in patients.

## Introduction

Obstructive sleep apnea (OSA) is characterized by intermittent narrowing or collapse of the airway during sleep^1^. Reopening of the airway after obstruction is often associated with electroencephalographic arousal. Arousal can attenuate apnea/hypopnea events but also causes sleep fragmentation^2^, which can result in excessive daytime sleepiness and cognitive impairment in OSA patients^3^. Moreover, propensity to arouse from sleep is a major physiological characteristic of OSA^4^.

The ascending reticular activating system (ARAS) is a network of brainstem structures involved in the maintenance of arousal and vigilance through its connection with cortical/subcortical regions^5^. Studies have suggested an association between the brainstem and OSA. In an animal model of OSA, the parabrachial complex was associated with respiratory-related arousal^5^. OSA patients showed altered brainstem structure and function associated with sympathetic activity^6^ and changes in brainstem regional homogeneity associated with OSA severity^7^. Patients with OSA may have a neural arousal-associated pattern generator that reacts to an obstructive respiratory event^8^.

Resting-state functional magnetic resonance imaging (fMRI) can detect functional connectivity (FC) between different brain areas reliably^9^. Previous studies have evaluated ARAS–cortex FC using resting-state fMRI in healthy populations^10^, patients with temporal lobe epilepsy^11^, and patients with chronic insomnia disorder^12^. Another study showed that widespread ARAS–cortex FC values are affected by age^13^. fMRI may be a useful approach to characterize the functional architecture between the ARAS brainstem structure and cerebral cortex/subcortex in OSA patients.

Repeated arousals or respiratory disturbances during sleep in OSA patients may be associated with changes in ARAS connectivity. However, no studies have evaluated ARAS–cortex FC in patients. We aimed to evaluate resting-state FC between eight ARAS nuclei and 105 cortical/subcortical regions in patients with moderate to severe OSA compared to healthy controls without OSA. Additionally, we evaluated the correlations of the extent of FC alterations with arousal and OSA severity among OSA patients.

## Results

### Patient characteristics

Fifty patients with moderate to severe OSA and twenty age- and sex-matched controls were analyzed. The mean arousal index in OSA patients was 45.3 ± 20.3 events/h with a mean apnea–hypopnea index (AHI) of 41.4 ± 20.1 events/h and a minimum oxygen saturation of 79.0 ± 8.9%. The patients showed higher Pittsburgh Sleep Quality Index (PSQI) scores and proportions of N1 sleep and lower proportions of N2 and N3 sleep than controls. They also had higher values of the arousal, apnea, hypopnea, and apnea–hypopnea indexes and lower minimum oxygen saturation than controls (Table 1).

**Table 1:** Patient characteristics.

	Control	Moderate to severe OSA	p-value
	n = 20	n = 50	
Age	44 ± 9	47 ± 10	0.183
Male (%)	14 (70.0)	49 (81.7)	0.269
BMI	25.6 ± 5.2	27.6 ± 4.4	0.137
HTN	1 (5.0)	15 (30.0)	0.024
DM	0	13 (26.0)	0.012
CVD	0	5 (10.0)	0.142
Sleep questionnaire			
PSQI	5.2 ± 3.3	9.0 ± 4.5	0.001
ESS	6.2 ± 2.6	7.6 ± 3.8	0.092
BDI	8.9 ± 5.5	8.5 ± 6.1	0.788
PSG results			
TST (min)	307.0 ± 81.3	283.1 ± 47.3	0.229
N1%	15.7 ± 11.4	32.9 ± 17.6	< 0.001
N2%	55.0 ± 10.6	40.2 ± 14.8	< 0.001
N3%	11.6 ± 8.1	5.7 ± 8.5	0.009
REM%	16.6 ± 7.7	13.7 ± 6.7	0.151
SE%	84.9 ± 20.2	81.7 ± 11.3	0.506
SL (min)	13.5 ± 21.6	7.8 ± 11.9	0.158
Arousal Index (/h)	14.3 ± 7.4	45.3 ± 20.3	< 0.001
Respiratory arousal (/h)	2.8 ± 2.3	35.5 ± 22.1	< 0.001
Spontaneous arousal (/h)	11.0 ± 6.6	8.2 ± 5.8	0.110
Apnea Index (/h)	0.4 ± 1.2	21.8 ± 21.7	< 0.001
Hypopnea Index (/h)	2.6 ± 2.2	19.6 ± 11.3	< 0.001
AHI (/h)	3.0 ± 2.3	41.4 ± 20.1	< 0.001
minSat%	89.3 ± 8.6	79.0 ± 8.9	< 0.001

The data are expressed as the mean ± standard deviation or number (percent).

*OSA* obstructive sleep apnea, *BMI* body mass index, *HTN* hypertension, *DM* diabetes mellitus, *CVD* cardiovascular disease, *PSQI* Pittsburgh Sleep Quality Index, *ESS* Epworth Sleepiness Scale, *BDI* beck depression inventory, *TST* total sleep time, *REM* rapid eye movement sleep, *SE* sleep efficiency, *SL* sleep latency, *AHI* apnea–hypopnea index, *minSat* minimum saturation.

### Differences in ARAS–cortex FC between controls and OSA patients

**Figure 1:**
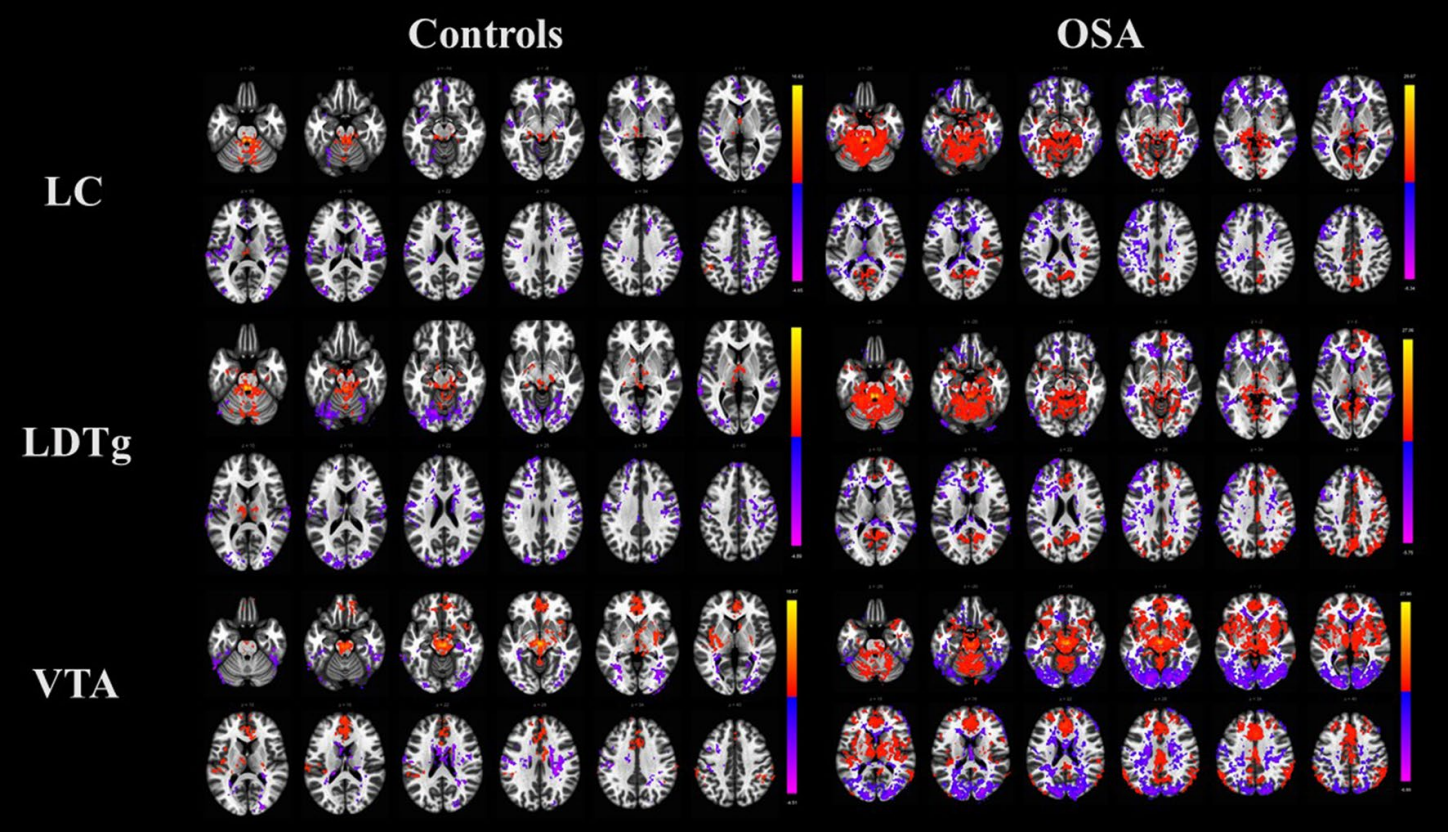
Functional connectivity of the LC, LDTg and VTA in healthy controls and OSA patients. Colors indicate brain areas that show positive (warm colors) and negative (cool colors) functional connectivity with three ARAS nuclei (the LC, LDTg, and VTA) according to a one-sample t test, *p* < 0.05, false discovery rate correction *p* < 0.05. *LC* locus coeruleus, *LDTg* laterodorsal tegmental nucleus, *VTA* ventral tegmental area, *ARAS* ascending reticular activating system.

Group differences in ARAS–cortex FC were observed within three ARAS nuclei: the locus coeruleus (LC), laterodorsal tegmental nucleus (LDTg), and ventral tegmental area (VTA). Voxel-wised FC t-maps of each ARAS nucleus in controls and OSA patients are presented in Fig. 1. controls exhibited negative FC of the LC with the precuneus and the posterior cingulate gyrus. In contrast, OSA patients exhibited positive FC of the LC with the precuneus and the posterior cingulate cortex. controls also exhibited a negative FC of the LDTg with the LOC and precuneus, whereas OSA patients showed positive connectivity of these regions. In controls, the VTA showed positive FC with the anterior cingulate gyrus and negative FC with the right LOC, similar to OSA patients.

**Figure 2:**
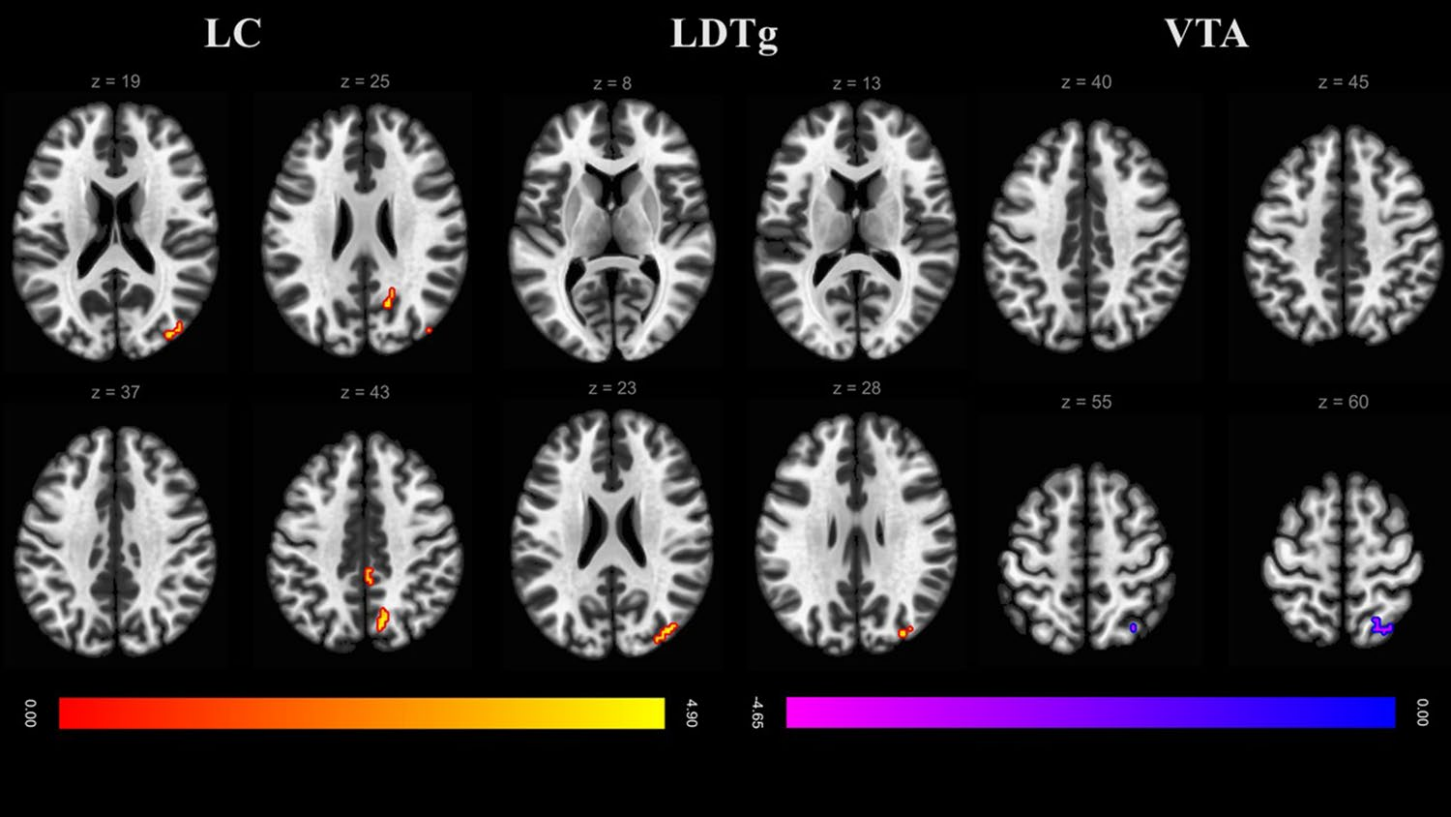
Axial views of differences in voxelwise functional connectivity between the ascending reticular activating system (ARAS) and the cortex between patients with moderate to severe OSA and healthy controls. Data represent t tests conducted on data from 50 OSA patients and 20 matched healthy controls (cluster-level *p* < 0.01, with false discovery rate correction *p* < 0.05). The OSA patients showed positive FC (warm colors) of the LC and LDTg and negative FC (cool colors) of the VTA to a greater extent than controls. *LC* locus coeruleus, *LDTg* laterodorsal tegmental nucleus, *VTA* ventral tegmental area.

Compared with the controls, OSA patients had stronger FC of the LC with the precuneus, right LOC, and posterior cingulate gyrus. Additionally, OSA patients had stronger FC between the LDTg and right LOC than controls but weaker FC between the VTA and right LOC (Table 2 and Fig. 2).

**Table 2:** Between group difference in AAN functional connectivity (Seed to voxel connectivity adjusted for age).

Coordinates (MNI)			Peak region	Cluster size	Peak p (uncorr)	Size p (FDR)
x	y	z				
LC						
+ 12	− 66	+ 44	Precuneus cortex	36	0.000006	0.004443
+ 36	− 82	+ 20	Rt. lateral occipital cortex	29	0.000048	0.009006
+ 16	− 62	+ 26	Precuneus cortex	22	0.000022	0.027408
+ 04	− 38	+ 42	Cingulate gyrus, posterior	19	0.000096	0.041360
LDTg						
+ 38	− 78	+ 26	Rt. lateral occipital cortex	41	0.000035	0.001862
VTA						
+ 30	− 62	+ 56	rt. lateral occipital cortex	33	0.000016	0.005023

*LC* locus coeruleus, *LDTg* laterodorsal tegmental nucleus, *VTA* ventral tegmental area.

### Relationship with sleep parameters

**Figure 3:**
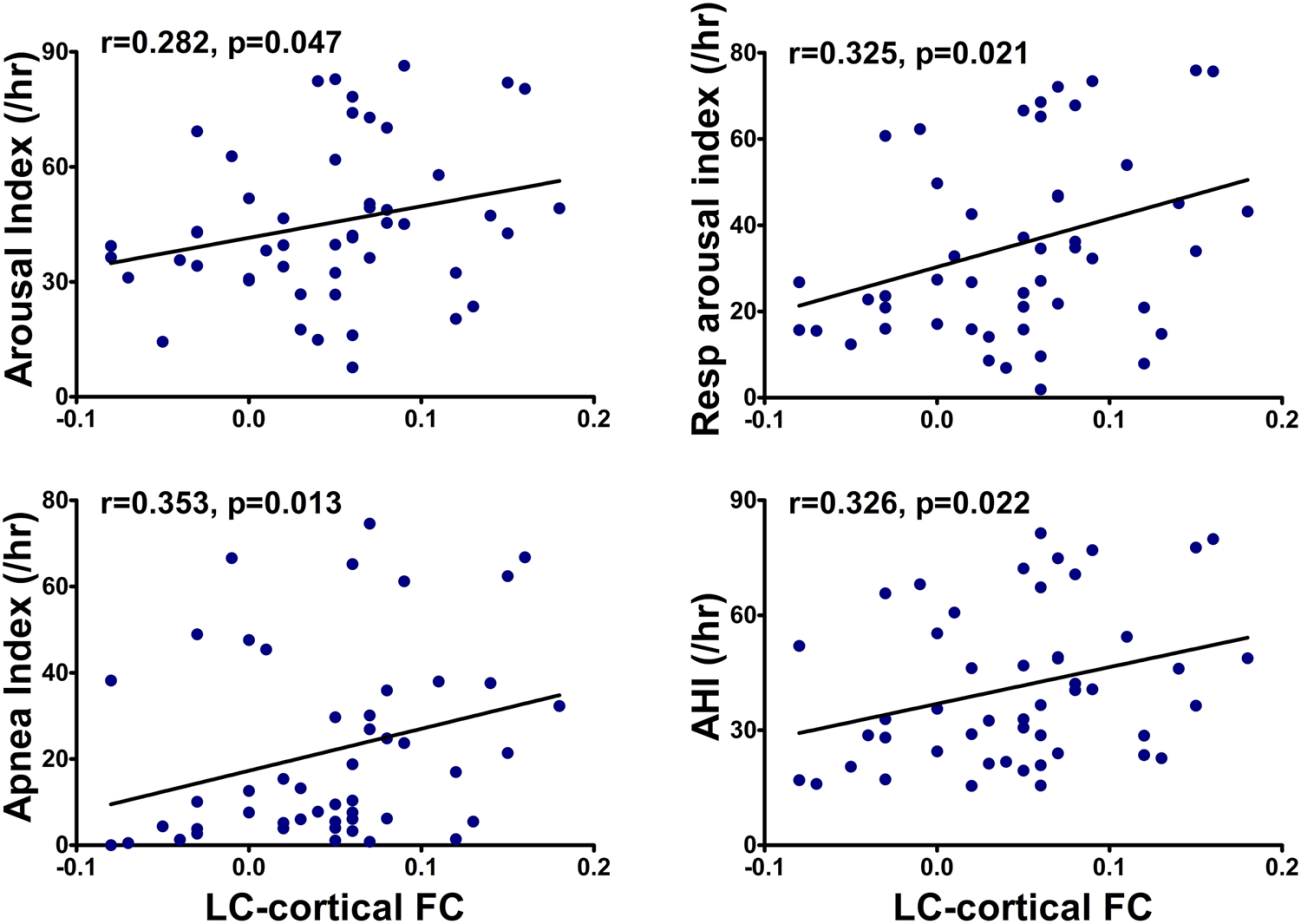
Correlation of FC between the LC and cortical regions with the arousal, apnea, and apnea–hypopnea indexes. Spearman’s correlation coefficient (rho) values showed positive correlations of LC–cortex FC with arousal, apnea, and apnea–hypopnea indexes. *LC* locus coeruleus, *FC* functional connectivity, *AHI* apnea–hypopnea index.

We next evaluated the correlation between alterations in ARAS–cortex FC in OSA patients and predefined polysomnography (PSG) parameters (values of the arousal index, respiratory arousal index, spontaneous arousal index, apnea index, hypopnea index, and AHI). Positive correlations were observed between altered LC–cortex FC and the arousal index (r = 0.282, p = 0.047), between altered LC–cortex FC and the respiratory arousal index (r = 0.325, p = 0.021), between altered LC–cortex FC and the apnea index (r = 0.353, p = 0.013), and between altered LC–cortex FC and the AHI (r = 0.326, p = 0.022) (Fig. 3). No significant correlations were observed between PSG parameters and FC value of the LDTg or VTA with the cortical regions.

## Discussion

We compared FC of eight ARAS nuclei with 105 cortical/subcortical areas between OSA patients and controls. Altered ARAS–cortex FC was observed in OSA patients within three ARAS nuclei: the LC, LDTg, and VTA. FC between the LC and cortical areas (the precuneus, posterior cingulate gyrus and right LOC) was stronger in OSA patients. FC between the LDTg and the right LOC was stronger in OSA patients compared to controls, but FC between the VTA and LOC was weaker. Only LC–cortex FC values were positively correlated with the arousal index, particularly respiratory arousals, the apnea index and AHI, in OSA patients.

FC can be defined as the synchronization between spatially remote neurophysiological events^14^. Therefore, functional connectivity refers to statistical dependence between the time series of electrophysiological activity and (de)oxygenated blood levels in distinct regions of the brain in the BOLD contrast. Functional connectivity can be altered in a patient with different diseases and has been reported to differ between patients and controls. The changes in FC of the LC, LDTg, and VTA with cortical regions in OSA patients may reflect altered monoaminergic modulation for maintaining arousal, specifically altered noradrenergic, cholinergic, and dopaminergic neuromodulation, respectively. The result is in line with an animal study with an OSA mouse model that showed loss of noradrenergic and dopaminergic neurons^15^, as in our study.

The LC is a noradrenergic region associated with various neurobiological processes, including sleep–wake modulation, attention facilitation, autonomic function, and the stress response^16,17^. A previous resting-state fMRI study of healthy adults revealed negative FC between the LC and the bilateral visual cortex, precuneus, and posterior cingulate cortex^10^, as we observed in controls. OSA patients, however, showed stronger FC between the LC and cortical areas in the default mode network (DMN; specifically, the precuneus and posterior cingulate cortex) and the right LOC. This result is in line with a previous study of patients with chronic insomnia who showed stronger FC between the LC and right precuneus or posterior cingulate cortex compared to controls^12^. Precuneus is associated with impaired consciousness in epilepsy, and a key region for spike-and-wave discharge in juvenile myoclonic epilepsy patients^18^. Increased FC between LC and areas involving precuneus may be a compensatory mechanism for sleep disturbance in OSA.

The LDTg is a cholinergic nucleus associated with arousal or emotional arousal under adverse conditions^19,20^. The VTA is a key structure that contains dopaminergic, GABAergic, and glutamatergic neurons and is associated with motivational processes^21^. Dopaminergic neurons in the VTA are key regulators of sleep and wakefulness^22^; however, the roles of GABAergic and glutamatergic neurons in the VTA are still unclear. In the present study, controls exhibited positive FC of the VTA with the anterior cingulate gyrus and negative FC of the VTA with the occipital cortex, which was in line with a previous VTA–cortex FC study in young adults^23^.

Regions with altered ARAS–cortex FC commonly included the right LOC, which is a key node for shape-object perception^24^. The LOC was suggested to play a causal role in the instantiation of the attentional filtering mechanism^25^. Several pharmacological studies have revealed topographical BOLD signal changes in the visual cortex following noradrenergic or cholinergic manipulation^26^. Structural and perfusion changes in the right LOC have been reported in patients with OSA^27,28^; however, their role should be further investigated.

The strength of FC between the LC and cortical areas including DMN (the precuneus and posterior cingulate gyrus) was positively associated with the severity of arousal or respiratory disturbance in OSA patients. The result suggests that noradrenergic transmission may play important role in mediating respiratory arousal and apnea events in OSA patients. Moreover, it may reveal the potential mechanism of pharmacotherapy for OSA. Recent studies showed that the noradrenergic agents, such as atomoxetine, combined with antimuscarinic agents can reduce the AHI, arousal index, and arousal threshold in OSA patients^29^.

The association of LC-cortical FC with arousal or AHI was consistent with previous studies. A pharmacological fMRI study showed that FC between the LC and posterior cingulate cortex is reduced during a dexmedetomidine-induced low arousal state and restored during the recovery state^30^. Moreover, increased FC between the LC and DMN regions in patients with chronic insomnia suggests that FC may be related to the level of arousal^12^. FC was associated with the apnea index and AHI, which is in line with a previous study that showed a correlation between brainstem regional homogeneity and OSA severity^7^. Changes in FC of noradrenergic regions in our study may also be associated with sympathetic hyperactivation in OSA patients^31^; similarly, muscle sympathetic nerve activity was coupled with BOLD signal changes in the brainstem^32^.

Several limitations should be considered when interpreting the results of the present study. This was a single-center study with a limited number of participants. However, we performed fMRI the following morning after PSG, which may more directly reflect the association between fMRI changes and PSG parameters. Moreover, although we enrolled patients with moderate to severe OSA who underwent fMRI in identical scanning conditions, heterogeneity in cognition or vigilance may have been present in these OSA patients, even among those with similar disease severity^33^. Comorbidities, including hypertension and diabetes, were more frequent in those with OSA, which may have affected the functional connectivity changes.

## Conclusions

These findings suggest that ARAS–cortex FC is altered in patients with moderate to severe OSA. Alteration of the LC-cortical noradrenergic FC may be associated with arousal and apnea–hypopnea severity in OSA. Future studies with larger numbers of patients are needed to elucidate the association between ARAS–cortex FC changes and OSA phenotypes such as arousal thresholds.

## Methods

### Participants

Consecutive patients with moderate to severe OSA (AHI ≥ 15 events/h) who visited Kyung Hee University Hospital in Gangdong were prospectively enrolled. Age- and sex-matched healthy volunteers were recruited online to serve as controls. These controls were screened for sleep-related symptoms and neurological or psychological diseases with a structured questionnaire and clinical interview. Those with sleep disorders (rapid eye movement sleep behavior disorder, restless legs syndrome, or periodic limb movement during sleep with a periodic limb movement index > 15/h) were excluded from the analysis after overnight video PSG. Individuals with structural abnormalities or neurological disease (e.g., stroke or neurodegenerative disease) were also excluded.

This study was approved by the Institutional Review Board of Kyung Hee University Hospital in Gangdong (IRB no: 2020-12-010). All methods were performed in accordance with the relevant guidelines and regulations of the institution. Written informed consent was obtained from all participants.

### Clinical assessment

Subjective sleep-related symptoms were evaluated using questionnaires prior to PSG. The questionnaire included the Pittsburgh Sleep Quality Index (PSQI)^34^, which evaluates sleep quality and architecture; the Epworth Sleepiness Scale (ESS)^35^, which evaluates daytime sleepiness, and the Beck Depression Inventory (BDI)-II^36^, which evaluates depression symptoms.

### Polysomnography

PSG was performed according to standard protocols using a digital polygraph system (Grass-Telefactor twin version 2.6, West Warwick, RI, USA). The PSG data were manually scored according to the *American Academy of Sleep Medicine (AASM) Manual for the Scoring of Sleep and Associated Events*, version 2.6^37^. Hypopnea was defined as a ≥ 30% drop in airflow, lasting ≥ 10 s, accompanied by ≥ 3% oxygen desaturation. Obstructive apnea was described as a continuous reduction in airflow ≥ 90% for ≥ 10 s along with evident respiratory effort. The AHI was defined as the sum of apnea and hypopnea events per hour during sleep.

### Image acquisition and preprocessing

Image acquisition and preprocessing was done as described in our previous study^38^. MRI was performed the morning following PSG using a 3.0 Tesla MRI system with a 32-channel encoding head coil (Ingenia, Philips Medical System, Best, The Netherlands). Resting-state fMRI was acquired with the following parameters: repetition time (TR) = 2,000 ms; echo time (TE) = 35 ms; field of view (FOV) = 220 × 220 mm; flip angle (FA) = 90°; acquisition voxel size = 3.3 × 3.3 × 3.3; reconstructed voxel size = 1.7 × 1.7 × 3.3 mm^3^; echo-planar imaging (EPI) factor = 33; and number of slices = 34. Participants were told to close their eyes and relax during the scan. They were interviewed afterwards whether they had fallen asleep during the scan.

Structural three-dimensional (3D) T1-weighted (T1W) images were acquired with the following parameters for image registration and segmentation: TR = 8.1 ms, TE = 3.7 ms, FA = 8°, FOV = 236 × 236 mm^2^, and voxel size = 1 × 1 × 1 mm^3^. To estimate for any structural abnormalities, T2-weighted turbo-spin‒echo fluid-attenuated inversion recovery (FLAIR) and gradient-echo images were also obtained.

All images were visually inspected by an independent radiologist. Participants with excessive head motion parameters (translation > 1.5 mm or rotation > 1.5° in any direction) were excluded. To avoid magnetic field saturation, the first 5 resting-state fMRI scans were discarded. We used Statistical Parametric Mapping (SPM) version 12 software (Wellcome Department of Cognitive Neurology, London, UK) and the CONN-fMRI FC toolbox (https://www.nitrc.org/projects/conn) version 21a^39^ to process the remaining images.

Default preprocessing pipeline in the CONN toolbox was used for preprocessing the remaining 175 functional images; each subject’s functional images were realigned to the first volume, unwarped, slice-timing corrected (interleaved bottom-up), coregistered with structural data, and spatially normalized into the standard MNI space. A spatial smoothing with a 2-mm full-width half-maximum (FWHM) isotropic Gaussian kernel was applied. A component-based noise correction method (CompCor)^40^ was used to regress out and correct for head motion, physiological noise, and nuisance signals, including six motion parameters, signals from white matter, and cerebrospinal fluid (CSF) voxels. There was no significant difference in six motion realignment parameters (controls: 0.017 ± 0.058 vs. OSA patients: 0.016 ± 0.050, p = 0.442) or global signal change and framewise displacement (controls: 0.478 ± 0.043 vs. OSA patients: 0.497 ± 0.039, P = 0.105). Then, bandpass of 0.009–0.08 Hz filter was applied and the signal was linearly detrended.

### Region of interest seed region

**Figure 4:**
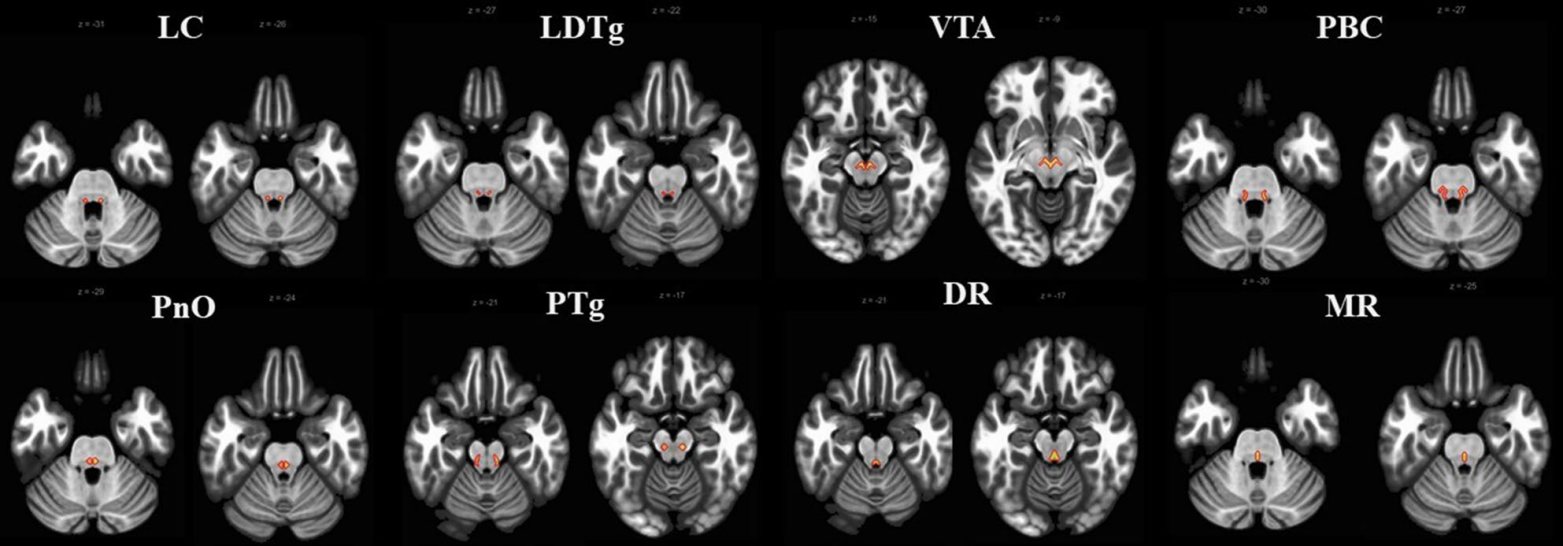
Axial slices of eight nuclei from the Harvard Ascending Arousal Network Atlas overlaid on brain anatomy (grayscale). Each nucleus was used as a seed for fMRI analysis. *LC* locus coeruleus, *LDTg* laterodorsal tegmental nucleus, *VTA* ventral tegmental area, *PBC* parabrachial complex, *PnO* pontine nucleus oralis, *PTg* pedunculopontine tegmental nucleus, *DR* dorsal raphe nucleus, *MR* median raphe nucleus.

ARAS structures from the Harvard Ascending Arousal Network Atlas provided by the Martinos Center for Biomedical Imaging (Charleston, Massachusetts, USA, https://www.nmr.mgh.harvard.edu/resources/aan-atlas)^41^ were used to identify the regions of interest (ROIs) consisting of the eight ARAS nuclei (Fig. 4). The atlas used high-resolution diffusion tensor imaging with postmortem histologic examination of the human brain to determine MNI coordinates of ARAS nuclei. The nuclei included the LC, LDTg, VTA, parabrachial complex (PBC), pontine nucleus oralis (PnO), pedunculopontine tegmental nucleus (PTg), and dorsal and median raphe nucleus (DR and MR, respectively).

### Seed-to-voxel functional connectivity analysis

Second-level seed-to-voxel analysis was performed to measure FC between each ARAS nucleus and 105 cortical/subcortical regions excluding cerebellar regions in the Harvard–Oxford atlas in the CONN toolbox^42^. The temporal correlations between the blood oxygen level-dependent (BOLD) signals of each ARAS nucleus and other voxels in the whole brain were computed with partial Pearson’s correlation analyses. A generalized linear model (GLM) one-way analysis of variance (ANOVA) was conducted to evaluate the difference in FC patterns between the groups, with age as a covariate. To identify significant clusters, an uncorrected peak value threshold of a p value < 0.001 and a familywise error (FWE)-corrected threshold of a p value < 0.05 were applied after adjusting for age.

### Statistical analysis

Continuous data were tested for normality of distribution with the Kolmogorov–Smirnov test and are presented as the mean ± standard deviation (SD). Two-sample t tests or Mann–Whitney U tests, as appropriate, were used to evaluate differences in demographic and PSG parameters between OSA patients and controls. Among OSA patients, Spearman’s rank correlation coefficient (rho) values were calculated to assess the nonparametric association between aberrant ARAS–cortex FC values and sleep parameters (values of the arousal index, apnea index, hypopnea index, and AHI) in OSA patients. The significance level was set to 0.05. All statistical comparisons were conducted using SPSS (version 22.0, Chicago, IL, USA).

## Data Availability

Data are available from the corresponding author upon reasonable request.
